# Supplementary material for: Diet and BMI Correlate with Metabolite Patterns Associated with Aggressive Prostate Cancer
Source: Nutrients. 2022 Aug 12;14(16):3306. doi: 10.3390/nu14163306 (PMC9415102; doi:10.3390/nu14163306)
Supplement: Supplementary file 1 [file nutrients-14-03306-s001.zip › nutrients-1847309-supplementary.pdf]

Table S1. Instruments used to quantify metabolites in the study population		
**Method**	**Instrument**	**Study**
Triple quadrupole mass spectrometry (MS)	Triple Quad 4500, AB Sciex, Framingham, MA	Prostate<br>Colorectal
	Q-Trap 5500, AB Sciex, Framingham, MA	Liver<br>Kidney
Liquid chromatography (LC)	Agilent 1290, Agilent Technologies, Santa Clara, CA	Prostate<br>Colorectal<br>Liver<br>Kidney

Table S2. Associations of dietary exposures and BMI with metabolite patterns in the discovery and validation sets.

		Discovery			Validation	
**Metabolite pattern**	**Exposure**	**β**	**P-value**	**P_{adj}**	**β**	**P-value**
1	**Alcohol**	**0.061**	**8.0E-11**	**1.9E-09**	**0.044**	**3.5E-02**
	Butter	0.047	2.4E-06	2.9E-05	0.026	2.6E-01
	**Total fish**	**0.076**	**4.2E-06**	**3.3E-05**	**0.12**	**3.8E-04**
	**Total fish products**	**0.067**	**1.0E-05**	**4.8E-05**	**0.12**	**3.6E-04**
	Margarine	-0.033	1.0E-05	4.8E-05	-0.0097	5.8E-01
	Fatty fish	0.043	4.5E-04	1.8E-03	0.049	7.9E-02
	**Lean fish**	**0.026**	**1.0E-03**	**3.5E-03**	**0.044**	**8.7E-03**
	Cereals	-0.11	3.2E-03	9.5E-03	-0.10	1.2E-01
	BMI	-0.015	1.5E-02	4.1E-02	0.0088	5.2E-01
	Dairy	0.041	3.4E-02	8.1E-02		
	Cheese	0.035	6.9E-02	1.5E-01		
	Red meat	0.040	8.4E-02	1.7E-01		
	Milk	0.034	1.1E-01	2.0E-01		
	Vegetables (fruiting)	0.046	2.1E-01	3.7E-01		
	Yogurt	0.011	2.3E-01	3.7E-01		
	Fruit	0.011	3.1E-01	4.7E-01		
	Eggs	-0.0055	5.1E-01	7.2E-01		
	Vegetable oils	0.0044	5.7E-01	7.2E-01		
	Vegetables	0.0092	5.6E-01	7.2E-01		
	Vegetables (leafy)	-0.0055	6.2E-01	7.4E-01		
	Fats	-0.0042	7.6E-01	8.6E-01		
	Vegetables (root)	-0.0060	7.9E-01	8.6E-01		
	Poultry	-0.0038	8.5E-01	8.8E-01		
	Processed meat	0.0022	9.1E-01	9.1E-01		
2	**BMI**	**0.039**	**5.9E-11**	**1.4E-09**	**0.046**	**1.0E-03**
	Butter	-0.049	5.4E-07	6.4E-06	-0.046	5.5E-02
	Dairy	-0.078	5.0E-05	3.6E-04	-0.057	1.7E-01
	Margarine	0.030	6.0E-05	3.6E-04	0.0070	6.9E-01
	Milk	-0.073	4.8E-04	2.3E-03	-0.051	2.6E-01
	Alcohol	0.029	1.8E-03	7.3E-03	-0.015	4.8E-01
	Cereals	0.090	1.3E-02	4.3E-02	0.11	1.0E-01
	Processed meat	0.028	1.6E-01	4.3E-01		
	Fats	0.019	1.5E-01	4.3E-01		
	Yogurt	-0.010	2.4E-01	5.0E-01		
	Poultry	0.024	2.2E-01	5.0E-01		
	Vegetable oils	0.0086	2.5E-01	5.0E-01		
	Vegetables (leafy)	-0.012	2.8E-01	5.2E-01		
	Cheese	0.016	4.1E-01	6.1E-01		
	Total fish products	-0.013	3.9E-01	6.1E-01		
	Total fish	-0.013	4.4E-01	6.1E-01		
	Lean fish	-0.0060	4.5E-01	6.1E-01		
	Vegetables	-0.013	4.0E-01	6.1E-01		
	Vegetables (fruiting)	-0.023	5.4E-01	6.4E-01		
	Fruit	0.0066	5.3E-01	6.4E-01		
	Fatty fish	-0.0067	5.9E-01	6.7E-01		
	Vegetables (root)	0.0080	7.2E-01	7.8E-01		
	Red meat	0.0052	8.2E-01	8.6E-01		
	Eggs	-0.0011	8.9E-01	8.9E-01		
3	**BMI**	**-0.068**	**5.7E-32**	**1.4E-30**	**-0.036**	**7.4E-03**
	Alcohol	0.044	6.9E-07	8.3E-06	0.025	2.2E-01
	**Fatty fish**	**-0.041**	**4.1E-04**	**3.3E-03**	**-0.071**	**9.7E-03**
	Total fish products	-0.041	4.1E-03	2.4E-02	-0.026	4.2E-01
	Eggs	0.021	8.9E-03	3.8E-02	0.015	4.4E-01
	Total fish	-0.041	9.6E-03	3.8E-02	-0.028	4.3E-01

	Butter	0.016	8.1E-02	2.8E-01
	Margarine	-0.011	1.2E-01	3.2E-01
	Vegetables (fruiting)	0.055	1.2E-01	3.2E-01
	Cereals	-0.050	1.5E-01	3.6E-01
	Milk	0.026	1.9E-01	3.9E-01
	Poultry	-0.025	1.8E-01	3.9E-01
	Dairy	0.021	2.5E-01	4.3E-01
	Lean fish	-0.0085	2.4E-01	4.3E-01
	Cheese	0.015	4.1E-01	5.4E-01
	Processed meat	0.016	4.1E-01	5.4E-01
	Vegetables	0.013	3.9E-01	5.4E-01
	Vegetables (leafy)	0.0098	3.5E-01	5.4E-01
	Vegetables (root)	-0.016	4.4E-01	5.6E-01
	Red meat	0.0084	7.1E-01	8.2E-01
	Vegetable oils	0.0026	7.2E-01	8.2E-01
	Yogurt	-0.0022	8.0E-01	8.7E-01
	Fats	-0.0014	9.1E-01	9.1E-01
	Fruit	0.0016	8.8E-01	9.1E-01

All models were adjusted for age at blood collection, time of day of blood collection, energy intake (kcal/day), fasting status at blood collection, education level, physical activity, smoking status, baseline alcohol consumption, and body mass index (BMI). Models that examined alcohol intake and BMI as main exposures were not adjusted for alcohol intake and BMI, respectively.

Variables are ordered by lowest P_{adj} value per metabolite pattern.

To account for multiple testing, only dietary correlates of treelets that passed the false discovery rate of 0.05 ($P_{adj} < 0.05$), using the Benjamini-Hochberg method, were further analysed in the validation set.

Bolded variables within the table refer to exposures that were found to be significant (P-value < 0.05) in the validation set.

β is per 1 unit described in Table 1 (excluding BMI, in which β is per 1 unit kg/m²).

Abbreviations: BMI, body mass index

Table S3. Associations of validated dietary exposures and BMI with individual metabolites loading on each metabolite pattern					
Metabolite pattern	Exposure	Metabolite	β	P-value	P_{adj}
1	Alcohol	PC aa C32:1	0.082	5.8E-91	1.5E-88
	Alcohol	PC aa C34:1	0.038	4.1E-68	5.5E-66
	Alcohol	PC aa C36:4	0.029	3.5E-53	3.1E-51
	Total fish products	PC aa C42:2	0.048	1.1E-47	7.0E-46
	Total fish	PC aa C42:2	0.052	1.5E-46	8.3E-45
	Alcohol	PC ae C36:0	0.030	2.6E-46	1.2E-44
	Total fish products	PC aa C38:0	0.049	3.1E-46	1.2E-44
	Alcohol	PC aa C32:0	0.023	9.9E-46	3.3E-44
	Total fish	PC aa C38:0	0.052	8.6E-44	2.6E-42
	Total fish products	PC aa C38:6	0.054	3.7E-43	9.9E-42
	Alcohol	PC ae C36:2	-0.026	1.9E-41	4.7E-40
	Total fish	PC aa C38:6	0.057	2.2E-39	4.8E-38
	Total fish products	PC aa C36:0	0.050	2.1E-38	4.4E-37
	Total fish	PC aa C36:0	0.054	4.0E-36	7.6E-35
	Alcohol	PC aa C36:5	0.049	2.6E-32	4.7E-31
	Total fish products	PC aa C40:6	0.049	1.1E-31	1.9E-30
	Alcohol	SM(OH) C16:1	-0.023	7.8E-29	1.2E-27
	Total fish products	PC aa C36:6	0.057	9.7E-29	1.4E-27
	Total fish	PC aa C40:6	0.052	9.5E-29	1.4E-27
	Alcohol	PC aa C30:0	0.029	1.4E-28	1.7E-27
	Alcohol	PC aa C34:4	0.030	1.3E-28	1.7E-27
	Total fish products	PC aa C36:5	0.071	4.7E-28	5.7E-27
	Total fish products	PC ae C40:6	0.034	7.1E-28	8.2E-27
	Total fish	PC aa C36:6	0.061	1.8E-27	2.0E-26
	Total fish	PC ae C40:6	0.037	3.3E-27	3.5E-26
	Total fish	PC aa C36:5	0.076	4.8E-27	5.0E-26
	Alcohol	PC ae C36:5	0.022	6.1E-27	6.1E-26
	Lean fish	PC aa C42:2	0.018	1.9E-26	1.9E-25
	Alcohol	SM(OH) C14:1	-0.022	1.1E-25	1.0E-24
	Alcohol	PC aa C36:1	0.024	1.4E-25	1.2E-24
	Total fish products	PC ae C38:6	0.034	1.8E-25	1.6E-24
	Alcohol	SM(OH) C22:2	-0.019	5.5E-25	4.6E-24
	Alcohol	PC aa C38:5	0.022	7.0E-25	5.7E-24
	Total fish	PC ae C42:3	0.032	9.5E-25	7.5E-24
	Total fish products	PC ae C36:0	0.033	3.1E-24	2.4E-23
	Total fish products	PC ae C42:3	0.029	3.3E-24	2.5E-23
	Total fish	PC ae C38:6	0.036	5.1E-24	3.7E-23
	Total fish	PC aa C42:0	0.036	2.6E-23	1.8E-22
	Total fish	PC aa C42:1	0.036	4.0E-23	2.8E-22
	Total fish products	PC aa C42:0	0.033	1.9E-22	1.2E-21
	Total fish	PC ae C36:0	0.035	2.9E-22	1.9E-21
	Total fish products	PC aa C42:1	0.032	3.6E-22	2.3E-21
	Alcohol	PC aa C34:3	0.021	4.2E-21	2.6E-20
	Alcohol	PC aa C40:4	0.020	3.1E-20	1.9E-19
	Alcohol	PC ae C30:2	-0.017	4.9E-19	2.9E-18
	Alcohol	PC ae C38:3	-0.016	1.0E-18	5.8E-18
	Alcohol	PC aa C38:6	0.021	3.0E-17	1.7E-16
	Lean fish	PC aa C38:0	0.015	5.7E-17	3.2E-16
	Total fish products	PC aa C40:4	-0.029	6.3E-17	3.4E-16
	Alcohol	PC aa C40:5	0.019	3.1E-16	1.6E-15
	Total fish	PC aa C40:4	-0.030	2.4E-15	1.3E-14
	Total fish products	PC ae C40:1	0.024	3.0E-15	1.5E-14
	Total fish	PC ae C40:1	0.027	3.0E-15	1.5E-14
	Total fish products	PC ae C42:2	0.024	3.4E-15	1.7E-14
	Total fish	PC ae C42:2	0.026	6.1E-15	3.0E-14
	Total fish	PC ae C32:2	0.024	2.9E-14	1.4E-13
	Total fish products	PC ae C32:2	0.021	4.9E-14	2.3E-13
	Total fish products	PC aa C40:2	0.027	9.2E-14	4.2E-13
	Alcohol	PC aa C36:6	0.024	9.7E-14	4.4E-13
	Lean fish	PC aa C36:0	0.015	2.4E-13	1.1E-12
	Lean fish	PC ae C36:0	0.012	1.2E-12	5.3E-12
	Alcohol	PC aa C38:4	0.015	2.3E-12	1.0E-11
	Total fish	PC aa C40:2	0.028	2.5E-12	1.1E-11

Alcohol	PC aa C34:2	0.0090	4.0E-12	1.7E-11
Total fish products	PC ae C40:2	0.022	4.1E-12	1.7E-11
Lean fish	PC ae C42:3	0.010	4.9E-12	2.0E-11
Total fish	PC ae C40:2	0.024	7.7E-12	3.1E-11
Total fish products	PC ae C38:4	-0.019	8.2E-12	3.2E-11
Lean fish	PC aa C38:6	0.014	1.2E-11	4.8E-11
Alcohol	PC ae C40:6	-0.013	1.7E-11	6.5E-11
Lean fish	PC ae C40:6	0.011	7.7E-11	2.9E-10
Lean fish	PC aa C42:1	0.011	9.2E-11	3.4E-10
Alcohol	PC ae C40:1	0.012	2.8E-10	1.0E-09
Total fish	PC ae C38:4	-0.019	4.1E-10	1.5E-09
Alcohol	PC ae C32:1	0.010	9.0E-10	3.2E-09
Alcohol	PC ae C34:2	-0.011	1.0E-09	3.6E-09
Alcohol	PC aa C36:3	0.010	1.1E-09	3.8E-09
Lean fish	PC ae C32:2	0.0090	1.5E-09	5.1E-09
Alcohol	PC ae C40:3	-0.0090	1.8E-09	6.0E-09
Lean fish	PC ae C38:6	0.010	3.1E-09	1.1E-08
Alcohol	PC aa C32:3	-0.011	4.6E-09	1.5E-08
Lean fish	PC aa C40:6	0.013	5.0E-09	1.6E-08
Alcohol	PC ae C38:2	-0.010	1.9E-08	6.1E-08
Alcohol	PC ae C42:1	0.0090	1.9E-08	6.1E-08
Lean fish	PC aa C36:6	0.015	2.4E-08	7.5E-08
Lean fish	PC ae C40:1	0.0090	2.4E-08	7.5E-08
Lean fish	PC aa C42:0	0.010	2.7E-08	8.3E-08
Lean fish	PC aa C36:5	0.019	3.6E-08	1.1E-07
Total fish products	PC aa C40:3	0.019	6.3E-08	1.9E-07
Total fish products	PC aa C38:4	-0.018	8.7E-08	2.6E-07
Lean fish	PC ae C42:2	0.0080	9.6E-08	2.8E-07
Alcohol	PC aa C42:2	0.011	1.4E-07	4.0E-07
Total fish	PC aa C38:4	-0.019	1.7E-07	4.9E-07
Total fish	PC aa C40:3	0.020	2.7E-07	7.7E-07
Total fish	PC ae C34:0	0.020	5.5E-07	1.5E-06
Alcohol	PC ae C36:4	0.010	6.2E-07	1.7E-06
Alcohol	PC ae C32:2	0.0090	1.4E-06	3.8E-06
Total fish products	PC aa C36:4	-0.014	1.5E-06	4.1E-06
Lean fish	PC ae C40:2	0.0080	2.4E-06	6.5E-06
Total fish products	PC ae C34:0	0.017	4.2E-06	1.1E-05
Total fish	PC aa C36:4	-0.015	4.6E-06	1.2E-05
Total fish products	PC aa C42:5	0.017	1.0E-05	2.5E-05
Total fish products	PC ae C40:4	-0.011	1.0E-05	2.5E-05
Lean fish	PC aa C40:2	0.0081	1.0E-05	2.5E-05
Lean fish	PC ae C34:0	0.0086	1.0E-05	2.5E-05
Alcohol	PC ae C38:6	0.0091	1.0E-05	2.5E-05
Alcohol	PC ae C38:5	0.0070	2.0E-05	5.0E-05
Alcohol	PC ae C40:4	-0.0068	2.0E-05	5.0E-05
Total fish	PC aa C42:5	0.017	3.0E-05	7.4E-05
Total fish	PC ae C30:2	0.013	4.0E-05	9.7E-05
Alcohol	PC aa C38:3	0.0083	4.0E-05	9.7E-05
Alcohol	PC aa C42:4	0.0072	7.0E-05	1.7E-04
Lean fish	PC ae C30:2	0.0060	9.0E-05	2.1E-04
Alcohol	PC aa C42:0	-0.0082	1.0E-04	2.4E-04
Total fish products	PC ae C36:4	-0.012	1.2E-04	2.8E-04
Total fish products	PC ae C30:2	0.011	1.3E-04	3.0E-04
Lean fish	PC ae C40:5	0.0050	1.4E-04	3.2E-04
Alcohol	PC ae C38:4	-0.0066	1.6E-04	3.6E-04
Total fish	PC ae C40:4	-0.010	2.0E-04	4.5E-04
Alcohol	PC ae C42:4	-0.0081	2.5E-04	5.6E-04
Total fish	PC aa C38:5	0.014	2.7E-04	5.9E-04
Alcohol	PC ae C42:5	-0.0058	2.7E-04	5.9E-04
Alcohol	PC aa C40:6	0.0097	2.8E-04	6.1E-04
Total fish products	PC aa C38:5	0.012	3.8E-04	8.2E-04
Total fish	PC ae C36:4	-0.012	4.0E-04	8.6E-04
Lean fish	PC aa C40:4	-0.0062	4.7E-04	1.0E-03
Total fish products	PC aa C36:3	-0.0094	5.1E-04	1.1E-03
Lean fish	PC aa C40:3	0.0061	5.6E-04	1.2E-03
Alcohol	PC ae C34:3	0.0073	8.5E-04	1.8E-03
Alcohol	PC ae C36:3	-0.0058	1.0E-03	2.1E-03

Total fish	PC aa C36:3	-0.0094	1.4E-03	2.9E-03
Total fish	SM(OH) C22:2	0.0099	1.8E-03	3.7E-03
Alcohol	PC aa C42:5	0.0073	1.8E-03	3.7E-03
Lean fish	SM(OH) C22:2	0.0047	1.8E-03	3.7E-03
Alcohol	PC aa C36:0	0.0076	2.1E-03	4.1E-03
Alcohol	PC ae C36:1	-0.0061	2.5E-03	4.8E-03
Alcohol	PC ae C42:2	0.0058	2.5E-03	4.9E-03
Lean fish	PC ae C30:0	0.0058	2.6E-03	5.0E-03
Total fish products	SM(OH) C22:2	0.0087	2.7E-03	5.3E-03
Alcohol	PC ae C30:0	-0.0069	3.1E-03	6.0E-03
Total fish	PC ae C30:0	0.012	3.6E-03	6.9E-03
Total fish	PC ae C38:2	0.0090	3.7E-03	6.9E-03
Total fish products	PC ae C42:4	-0.0102	4.0E-03	7.6E-03
Total fish products	PC aa C34:3	-0.010	4.5E-03	8.4E-03
Lean fish	PC ae C36:1	0.0048	4.9E-03	9.1E-03
Lean fish	PC aa C32:3	0.0042	5.2E-03	9.5E-03
Lean fish	PC ae C40:3	0.0035	5.7E-03	1.0E-02
Total fish	PC ae C44:6	0.010	5.8E-03	1.1E-02
Total fish	PC ae C40:5	0.0076	6.1E-03	1.1E-02
Lean fish	PC aa C36:4	-0.0041	6.1E-03	1.1E-02
Alcohol	PC ae C40:5	-0.0043	6.2E-03	1.1E-02
Total fish products	PC aa C40:5	-0.010	7.2E-03	1.3E-02
Total fish	PC ae C32:1	0.0078	7.6E-03	1.3E-02
Total fish products	PC ae C38:2	0.0075	7.9E-03	1.4E-02
Lean fish	PC ae C38:2	0.0039	8.0E-03	1.4E-02
Lean fish	PC aa C38:4	-0.0045	8.1E-03	1.4E-02
Total fish	PC aa C34:3	-0.0097	1.2E-02	2.1E-02
Total fish	PC aa C40:5	-0.010	1.2E-02	2.1E-02
Lean fish	PC ae C32:1	0.0035	1.3E-02	2.1E-02
Total fish products	PC aa C38:3	-0.0079	1.4E-02	2.4E-02
Lean fish	PC aa C28:1	.00412	1.5E-02	2.4E-02
Total fish products	PC ae C30:0	0.0091	1.6E-02	2.6E-02
Total fish	PC ae C36:1	0.0085	1.6E-02	2.6E-02
Total fish	PC aa C32:0	0.0068	1.7E-02	2.8E-02
Total fish	PC aa C28:1	0.0084	1.7E-02	2.8E-02
Total fish products	PC ae C40:5	0.0060	1.8E-02	2.8E-02
Alcohol	PC ae C40:2	-0.0047	1.8E-02	3.0E-02
Lean fish	PC ae C38:4	-0.0034	1.9E-02	3.0E-02
Lean fish	PC aa C42:5	0.0043	2.0E-02	3.1E-02
Total fish products	PC aa C28:1	0.0075	2.1E-02	3.3E-02
Lean Total fish	SM(OH) C14:1	0.0039	2.2E-02	3.4E-02
Total fish	PC aa C38:3	-0.0081	2.3E-02	3.6E-02
Total fish products	PC ae C32:1	0.0061	2.3E-02	3.6E-02
Alcohol	PC ae C42:3	0.0041	2.4E-02	3.7E-02
Total fish products	PC ae C44:6	0.0077	2.4E-02	3.7E-02
Lean fish	PC ae C42:1	0.0030	2.6E-02	3.9E-02
Total fish products	PC aa C34:4	-0.0097	2.6E-02	4.0E-02
Total fish products	PC ae C36:1	0.0071	2.7E-02	4.1E-02
Lean fish	PC aa C38:5	0.0038	3.3E-02	4.9E-02
Total fish products	PC aa C32:0	0.0054	3.7E-02	5.5E-02
Total fish	PC aa C32:3	0.0066	4.2E-02	6.2E-02
Total fish	PC ae C40:3	0.0054	4.5E-02	6.7E-02
Lean fish	SM(OH) C16:1	0.0033	4.6E-02	6.8E-02
Total fish	PC aa C34:4	-0.0094	4.9E-02	7.1E-02
Total fish	PC ae C42:4	-0.0076	5.1E-02	7.4E-02
Total fish products	PC aa C32:3	0.0057	5.3E-02	7.7E-02
Lean fish	PC ae C34:2	0.0029	5.6E-02	8.1E-02
Alcohol	PC aa C40:3	0.0041	6.3E-02	8.9E-02
Lean fish	PC ae C34:1	0.0027	6.8E-02	9.6E-02
Alcohol	PC aa C42:1	-0.0038	7.0E-02	9.9E-02
Total fish products	PC aa C42:4	-0.0052	7.4E-02	1.0E-01
Total fish products	PC ae C38:3	-0.0051	7.9E-02	1.1E-01
Lean fish	PC aa C36:1	0.0033	8.0E-02	1.1E-01
Total fish	SM(OH) C14:1	0.0064	8.1E-02	1.1E-01
Total fish products	PC aa C32:1	-0.011	8.5E-02	1.2E-01
Total fish products	PC ae C38:5	-0.0046	8.8E-02	1.2E-01
Lean fish	PC aa C34:1	0.0031	9.0E-02	1.2E-01

Total fish	SM(OH) C16:1	0.0059	9.9E-02	1.3E-01
Total fish	PC aa C42:4	-0.0051	1.1E-01	1.5E-01
Total fish products	PC ae C40:3	0.0039	1.1E-01	1.5E-01
Lean fish	PC ae C42:5	0.0021	1.2E-01	1.6E-01
Lean fish	PC ae C44:6	0.0027	1.2E-01	1.6E-01
Total fish	PC ae C38:5	-0.0045	1.2E-01	1.6E-01
Alcohol	PC ae C44:6	-0.0033	1.3E-01	1.7E-01
Alcohol	PC aa C36:2	0.0022	1.3E-01	1.7E-01
Lean fish	PC aa C34:3	-0.0027	1.3E-01	1.7E-01
Total fish	PC aa C32:1	-0.0102	1.4E-01	1.8E-01
Total fish	PC aa C34:1	0.0054	1.4E-01	1.8E-01
Total fish products	SM(OH) C16:1	0.0047	1.5E-01	1.9E-01
Lean fish	PC aa C34:4	-0.0032	1.5E-01	1.9E-01
Lean fish	PC ae C36:2	0.0022	1.5E-01	1.9E-01
Total fish products	PC aa C34:1	0.0048	1.5E-01	1.9E-01
Total fish	PC ae C42:1	0.0042	1.5E-01	1.9E-01
Total fish products	SM(OH) C14:1	0.0048	1.5E-01	1.9E-01
Lean fish	PC aa C32:0	0.0019	1.6E-01	2.0E-01
Alcohol	PC ae C44:5	0.0030	1.6E-01	2.0E-01
Total fish	PC ae C38:3	-0.0042	1.9E-01	2.3E-01
Lean fish	PC aa C40:5	-0.0025	1.9E-01	2.3E-01
Lean fish	PC ae C36:3	0.0018	2.1E-01	2.6E-01
Total fish products	PC aa C36:1	0.0044	2.2E-01	2.7E-01
Total fish	PC aa C36:1	0.0047	2.3E-01	2.8E-01
Total fish	PC ae C34:2	0.0038	2.3E-01	2.8E-01
Lean fish	PC ae C36:4	-0.0018	2.4E-01	2.8E-01
Total fish	PC ae C36:2	0.0038	2.5E-01	3.0E-01
Lean fish	PC ae C44:5	0.0020	2.7E-01	3.2E-01
Total fish	PC ae C36:5	0.0040	2.7E-01	3.2E-01
Lean fish	PC aa C32:1	-0.0034	3.0E-01	3.5E-01
Lean fish	PC ae C34:3	0.0018	3.1E-01	3.7E-01
Total fish products	PC ae C36:5	0.0033	3.3E-01	3.8E-01
Total fish products	PC ae C42:1	0.0026	3.3E-01	3.9E-01
Total fish products	PC ae C36:3	-0.0026	3.5E-01	4.1E-01
Alcohol	PC ae C34:0	0.0021	3.6E-01	4.1E-01
Lean fish	PC aa C42:4	-0.0013	3.8E-01	4.4E-01
Total fish products	PC ae C36:2	0.0026	3.9E-01	4.5E-01
Lean fish	PC aa C38:3	-0.0014	4.0E-01	4.6E-01
Lean fish	PC ae C36:5	0.0014	4.0E-01	4.6E-01
Lean fish	PC aa C36:3	-0.0012	4.1E-01	4.6E-01
Total fish products	PC ae C34:2	0.0024	4.2E-01	4.7E-01
Total fish	PC ae C34:3	0.0030	4.4E-01	5.0E-01
Alcohol	PC aa C38:0	-0.0016	4.5E-01	5.1E-01
Lean fish	PC ae C44:4	0.0012	4.7E-01	5.2E-01
Total fish products	PC aa C34:2	-0.0015	4.8E-01	5.3E-01
Lean fish	PC aa C34:2	-0.00080	4.8E-01	5.3E-01
Total fish	PC aa C34:2	-0.0016	4.9E-01	5.4E-01
Lean fish	PC ae C38:3	0.0010	4.9E-01	5.4E-01
Alcohol	PC ae C34:1	0.0012	5.1E-01	5.5E-01
Total fish	PC aa C30:0	0.0029	5.2E-01	5.7E-01
Alcohol	PC aa C40:2	-0.0014	5.5E-01	5.9E-01
Total fish	PC ae C34:1	0.0017	5.9E-01	6.4E-01
Total fish	PC ae C42:5	0.0015	6.0E-01	6.4E-01
Total fish	PC ae C36:3	-0.0015	6.3E-01	6.7E-01
Alcohol	PC ae C44:4	0.00090	6.3E-01	6.7E-01
Total fish	PC aa C36:2	-0.0012	6.5E-01	6.9E-01
Total fish products	PC aa C36:2	-0.0010	6.8E-01	7.2E-01
Total fish products	PC ae C44:4	-0.0012	6.9E-01	7.2E-01
Total fish products	PC aa C30:0	0.0012	7.8E-01	8.1E-01
Alcohol	PC aa C28:1	0.00060	7.8E-01	8.1E-01
Total fish products	PC ae C34:3	0.00090	8.0E-01	8.4E-01
Total fish	PC ae C44:5	0.00090	8.1E-01	8.4E-01
Total fish products	PC ae C44:5	-0.00080	8.2E-01	8.5E-01
Lean fish	PC ae C40:4	-0.00030	8.2E-01	8.5E-01
Lean fish	PC ae C38:5	0.00020	8.7E-01	8.9E-01
Lean fish	PC ae C42:4	0.00030	8.7E-01	8.9E-01
Total fish products	PC ae C34:1	0.00040	9.0E-01	9.1E-01

	Total fish	PC ae C44:4	0.00040	9.0E-01	9.1E-01
	Lean fish	PC aa C36:2	-0.00010	9.3E-01	9.4E-01
	Total fish products	PC ae C42:5	0.00020	9.5E-01	9.6E-01
	Lean fish	PC aa C30:0	0.00010	9.7E-01	9.7E-01
2	**BMI**	**Glutamate**	**0.030**	**1.1E-52**	**5.3E-52**
	BMI	C18:1	0.0027	9.3E-02	2.3E-01
	BMI	Ornithine	0.0017	2.3E-01	3.8E-01
	BMI	C18:2	-0.0015	4.6E-01	5.7E-01
	BMI	Taurine	0.00040	7.3E-01	7.3E-01
3	**BMI**	**Lyso PC a C18:1**	**-0.024**	**2.6E-58**	**4.2E-57**
	**BMI**	**Lyso PC a C18:2**	**-0.027**	**3.2E-57**	**2.5E-56**
	**BMI**	**Lyso PC a C17:0**	**-0.019**	**8.2E-35**	**4.4E-34**
	**BMI**	**Lyso PC a C18:0**	**-0.010**	**1.4E-17**	**5.8E-17**
	**BMI**	**Lyso PC a C20:4**	**-0.013**	**6.0E-17**	**1.9E-16**
	**BMI**	**Lyso PC a C16:0**	**-0.0081**	**8.6E-14**	**2.3E-13**
	**Fatty fish**	**Lyso PC a C20:4**	**-0.017**	**4.4E-08**	**1.0E-07**
	**Fatty fish**	**Lyso PC a C20:3**	**-0.017**	**1.6E-07**	**3.1E-07**
	**Fatty fish**	**Lyso PC a C16:1**	**-0.012**	**1.4E-04**	**2.5E-04**
	**Fatty fish**	**Lyso PC a C18:0**	**-0.0054**	**2.9E-02**	**3.5E-02**
	**Fatty fish**	**Lyso PC a C18:1**	**-0.0067**	**2.6E-02**	**3.5E-02**
	**BMI**	**Lyso PC a C16:1**	**-0.0034**	**2.7E-02**	**3.5E-02**
	**BMI**	**Lyso PC a C20:3**	**-0.0037**	**2.4E-02**	**3.5E-02**
	Fatty fish	Lyso PC a C16:0	-0.0044	4.8E-02	5.3E-02
	Fatty fish	Lyso PC a C17:0	-0.0061	5.3E-02	5.3E-02
	Fatty fish	Lyso PC a C18:2	-0.0064	5.3E-02	5.3E-02

All models were adjusted for age at blood collection, time of day of blood collection, energy intake (kcal), fasting status at blood collection, education level, physical activity, smoking status, baseline alcohol consumption, and body mass index (BMI). Models that examined alcohol intake and BMI as main exposures were not adjusted for alcohol intake and BMI, respectively.

Bolded variables within the table refer to exposures that passed the false discovery rate of 0.05 ($P_{adj} < 0.05$), using the Benjamini-Hochberg method.

Variables are ordered by lowest P_{adj} value

β is per 1 unit described in Table 1 (excluding BMI, in which β is per 1 unit kg/m²).

Abbreviations: BMI, body mass index
